# Acyl-*N*ω-methylserotonins and Branched-chain Acylserotonins in Lemon and Other Citrus Seeds—New Lipids with Antioxidant Properties and Potential Pharmacological Applications

**DOI:** 10.3390/biom12101528

**Published:** 2022-10-20

**Authors:** Jerzy Kruk, Agnieszka Trela-Makowej, Renata Szymańska

**Affiliations:** 1 Department of Plant Physiology and Biochemistry, Faculty of Biochemistry, Biophysics and Biotechnology, Jagiellonian University, Gronostajowa 7, 30-387 Kraków, Poland; 2 Faculty of Physics and Applied Computer Science, AGH University of Science and Technology, Reymonta 19, 30-059 Kraków, Poland

**Keywords:** acylserotonins, *Citrus limon*, Rutaceae, citrus species, branched fatty acids, plant lipids, seeds, antioxidant properties

## Abstract

We have found 15 previously unknown compounds in seeds of lemon and other citrus species, such as tangerine, grapefruit and pomelo. The structure of these compounds was characterized by HR–MS spectrometry, fluorescence spectroscopy and chemical synthesis. These compounds were predominantly long-chain (C20–C25), saturated acyl-*N*ω-methylserotonins with the main contribution of C22 and C24 homologues, usually accounting for about 40% and 30% of all acylserotonins, respectively. The other, previously undescribed, minor compounds were branched-chain acylserotonins, as well as normal-chain acylserotonins, recently found in baobab seed oil. Within the seed, acylserotonins were found nearly exclusively in the inner seed coat, where probably their biosynthesis proceeds. On the other hand, lemon seedlings contained only trace amounts of these compounds that were not found in adult leaves. The compounds identified in the present studies were shown to have antioxidant properties in vitro, using 2,2-diphenyl-1-picrylhydrazyl (DPPH) assay. In the investigated reaction in hexane, Me-C22 and Me-C24-serotonins were less active than n-C22 and n-C24-serotonins and δ-tocopherol, while branched-chain acylserotonins (iso-C21 and -C25) showed higher antioxidant activity than all the normal-chain compounds. On the other hand, all these compounds showed a similar but considerably lower antioxidant activity in acetonitrile than in hexane.

## 1. Introduction

**Figure 1:**
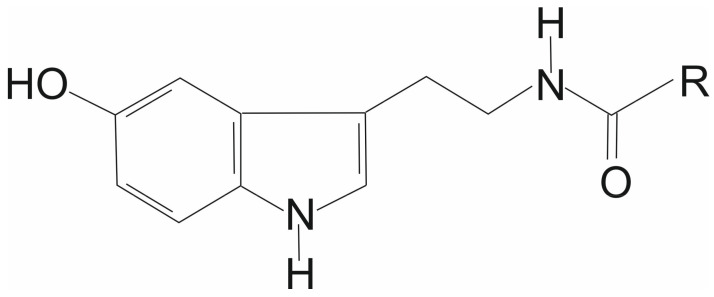
Chemical structure of *N*-acylserotonins. R—fatty acid side-chain.

Plant seeds and seed oils are a source of various compounds beneficial for human health, such as tocopherols and plastochromanol, showing antioxidant function [1,2,3] or phytosterols known for their anticholesterolomic effects [4,5]. Besides these compounds, there are also less widespread or unique antioxidants in plant seed oils, such as tocotrienols (mainly palm seed oil, rice bran oil) [1], γ-oryzanol [6], desmethyltocotrienols (rice bran oil) [7] or *N*-ferulylserotonin (safflower oil) [8], whose additional physiological and pharmacological functions have been shown [7,9,10]. Moreover, we have recently found a previously unknown class of lipids, *N*-acylserotonins in baobab seed oil and seeds, with mainly C22 to C26 fatty acid side-chains (Figure 1) [11]. These compounds showed antioxidant properties in model systems, similar to those of tocopherols [11]. 

During analysis of the lipid components of lemon seeds, we have found a number of unidentified fluorescent compounds. Therefore, we focused on the isolation of these compounds, determination of their chemical structure and occurrence in seeds of various lemon varieties and other citrus species. Moreover, as serotonin is known to have antioxidant properties, the selected identified compounds were investigated for their antioxidant potential in vitro.

## 2. Materials and Methods

### 2.1. Plant Material and Chemicals

Citrus fruits were obtained from local markets. Unless otherwise stated, seeds from *Citrus limon* (L.) Burm. ‘Lamas’ (country of origin—Turkey) were used for experiments. Other citrus fruits analyzed were of *Citrus limon* ‘Primaflora’ (Italy) and ‘Verna’ (Spain), tangerine (*Citrus reticulate* Blanco), grapefruit (*Citrus paradisi* Macfad.) and pomelo (*Citrus maxima* (Burm. f. Merr). Lemon seed oil (country of origin—Italy) was from Manufaktura Natura (Zgłobice, Poland).). 

Organic solvents were of HPLC grade (Sigma–Aldrich, Darmstadt, Germany; Bujno Chemicals, Poznań, Poland) unless otherwise stated. Chloroform used for preparative column chromatography was stabilized with amylene (~50 ppb). Serotonin, serotonin hydrochloride, 2-methyl-serotonin maleate, α-methylserotonin maleate, *N*ω-methyl-5-hydroxytryptamine oxalate (*N*ω-methylserotonin), EDC (*N*-(3-dimethylaminopropyl)-*N*′-ethylcarbodiimide hydrochloride), triethylamine, anhydrous tetrahydrofuran (THF), dimethylformamid (DMF), behenic, tricosanoic, lignoceric and hexacosanoic acids were *N*ω-methylserotonin from Sigma–Aldrich (Darmstadt, Germany. HOBT (1-hydroxybenzotriazole hydrate) was from Pol-Aura (Olsztyn, Poland). Pentacosanoic acid was from Santa Cruz Biotechnology (Dallas, TX, USA) while heptacosanoic acid was from TCI Chemicals (Tokyo, Japan). *Iso* fatty acids: 23-Methyltetracosanoic acid (*iso*-C25), and 19-Methyleicosanoic acid (iso-C21) were from Larodan AB (Malmo, Sweden). *Anteiso* fatty acids: 18-Methyleicosanoic acid [ 18-MEA ](ai-C21), 20-Methyldocosanoic acid (ai-C23), 22-Methyltetracosanoic acid (ai-C25) and 24-Methylhexacosanoic acid (ai-C27) were obtained from Nacalai (Kyoto, Japan). Alumina N used for column chromatography (activity grade I) was from MP Biomedicals (Eschwege, Germany).

### 2.2. Extraction and Purification Procedure

For analytical purposes, whole fresh seeds or their separated components (outer, inner seed coats, endosperm) were extracted with methanol or the HPLC solvent (ACN/Me/H_2_O, 72/8/1, *v*/*v*)by grinding in a mortar (Equimed, Krakow, Poland). The methanol extract was then evaporated to dryness in a stream of nitrogen, dissolved in 0.3–1.2 mL of the HPLC solvent, shortly centrifuged and subject to the HPLC system. When the HPLC solvent was used for the extraction, the extract after centrifugation was directly analyzed by HPLC.

For preparative isolation of the individual compounds and their further structural analysis, inner seed coats (1.07 g FW) from 130 lemon seeds (obtained from 3.8 kg of ‘Lamas’ variety), mechanically separated from the outer coats using precise tweezers (Topex, Poland), were ground in mortar first with liquid nitrogen to fine powder and then twice with methanol (2 × 25 mL). The methanol extracts were collected and the sediment was again extracted twice with the HPLC solvent (2 × 25 mL). The extracts were combined, evaporated on a rotary evaporator (BÜCHI Rotavapor R-300; Mainz, Germany)) dissolved in 40 mL chloroform (stabilized with 1% ethanol)and subject to column chromatography on Alumina N column (deactivated to grade III, 150 mL volume, 30 mm i.d.). The column was developed first in chloroform and then the investigated compounds were eluted with 12.5% and 25% methanol in chloroform. The pooled methanol/chloroform fractions were evaporated, dissolved in 5 mL of the HPLC solvent and subject to preparative HPLC to collect the fluorescent compounds. The HPLC purification of the fluorescent compounds was repeated twice or three times to remove the impurities.

For both analytical and preparative purposes, HPLC was performed using Nucleosil 100 C18 reverse-phase column (MZ Analysentechnik, Mainz, Germany, 250 × 4 mm, 5 µm) and ACN/MeOH/H_2_O (72/8/1, *v*/*v*) at the flow rate of 1.5 mL/min as a solvent. The HPLC setup included Jasco PU-2080 Plus pump (Jasco, Tokyo, Japan) and Shimadzu RF-10 AXL fluorescence detector (MD, USA) (290/330 nm, excitation/emission The loop was 100 or 500 µL for analytical and preparative purposes, respectively.

### 2.3. Fluorescence Spectra 

Fluorescence excitation and emission spectra were recorded with Perkin–Elmer LS50B spectrofluorometer (Llantrisant, UK) in 1 × 1 cm fluorimetric cuvettes (Hellma Analytics, Müllheim, Germany), using 5 nm excitation and 5 nm emission slits. Excitation and emission wavelengths were 275 and 330 nm, respectively.

### 2.4. Mass Spectrometry

Mass spectrometry analyses were performed by direct injection of methanol solutions of isolated fractions using electrospray ion source in a positive mode and high-resolution tandem mass spectrometer Bruker Impact II (Bremen, Germany), equipped with a quadrupole time-of-flight mass analyzer (ESI-QTOF). The obtained *m*/*z* data were fitted to molecular formula using ChemCalc software (https://www.chemcalc.org accessed on 13 September 2022) and the following atom composition range C0-100 H0-200 N0-4 O0-20 Na1.

### 2.5. Synthesis of N-acylserotonins

*N*-acylserotonin standards were synthesized as described previously [11] with some modification. A fatty acid (0.1 mmol) was dissolved in a solution of 17.6 mg HOBT in 0.5 mL or 1 mL THF(for C24 or higher fatty acid homologues) and stirred with 19.3 mg EDC (22 µL) for 1 h at room temperature. Afterwards, solution of 24.1 mg serotonin hydrochloride in 300 µL or 600 µL DMF (for C24 or higher homologues) and 11.8 µL triethylamine were added, the mixture was shortly vortexed and stirred for 15 min at room temperature. The scheme of the synthesis is shown in Figure S1. The reaction mixture was then transferred to a 15 mL Falcon tube (Eppendorf, Warsaw, Poland), followed by addition of 5 ml ethyl acetateand ca. 5 mL water. The content of the tube was vigorously shaken for ca. 1 min, and after centrifugation (5000× *g* × 1 min), the upper organic phase was collected with a pipette and transferred to a rotary evaporation flask. The extraction with ethyl acetate was repeated and the combined organic phases were evaporated to dryness on a rotary evaporator. Then, the final product was dissolved in 3 mL of peroxide-free (freshly distilled), anhydrous THF. 

In the case of synthesis of *N*-acyl-*N*ω-methylserotonins, *N*ω-methyl-serotonin dissolved in 0.9 mL or 1.8 mL DMF (for C24 or higher homologues), was added dropwise to the reaction mixture under stirring. 

If the available amount of a fatty acid was low, the proportion of all the chemicals was scaled down, respectively. 

Concentration of *N*-acylserotonins in stock solutions was determined from absorption spectra in acetonitrile/methanol/water (72/8/1) using millimolar extinction coefficient of 6.37 at 276 nm in this solvent [11]. The identity of the synthesized *N*-acylserotonins was verified by mass spectrometry, absorption and fluorescence spectroscopy. 

### 2.6. Hydrolysis of N-acylserotonins and Lipids of Lemon Seed Oil 

Isolated fractions of compounds 5 and 9 were hydrolyzed in concentrated HCl/THF (1/9, *v*/*v*) for 1 h at 105 °C in tightly closed Wheaton 4 mL glass vials using thermoblock (Thermo Fischer Scientific, Shanghai, China. Afterwards, the content was evaporated to dryness in a stream of nitrogen and subsequently under vacuum. Then, the obtained fatty acids were coupled to serotonin as described above. 

About 100 mg of lemon seed oil was dissolved in 1 mL ethanol. Next, 100 µL of saturated NaOH)POCH, Gliwice, Poland) in water was added and the mixture was heated for 1 h at 90 °C using thermoblock (Eppendorf, Warsaw, Poland). Afterwards, the solution was neutralized with conc. HCl and the fatty acids were extracted with ethyl acetate after dilution the mixture with water. After evaporation under nitrogen, the obtained fatty acids were coupled to serotonin as described above.

### 2.7. Quantitative Determination of Individual Compounds in Seeds

The concentration of individual *N*-acylserotonins in the extracts was determined from the area of appropriate peaks in HPLC chromatograms and those of synthetic standards of known concentration. The content (in mg/g FW) was calculated based on the known weight of the seed samples and the volume of the solvent used for extraction. The extracts were prepared as described in Section 2.2.

### 2.8. Determination of the Reaction Rate Constants with DPPH

Determination of the antioxidant-DPPH reaction rate constants was performed as described previously [11] under pseudo-first order conditions, i.e., the excess of one of the reaction substrates, according to [12,13]. The initial concentrations of DPPH and the investigated antioxidants in the reaction mixture were 75 µM and 7.5 µM, respectively. Stock solutions used were 5 mM DPPH in methanol, 2 mM of tocopherols in ethanol and 3–6 mM *N*-acylserotonins in THF. Appropriate volume of the solution of the investigated compound was rapidly injected into 2ml of DPPH solution in acetonitrile or hexane under continuous stirring at room temperature. Bleaching of DPPH was followed spectrophotometrically at 520 nm, using Jasco V-650 spectrophotometer (Tokyo, Japan).. The half-time of the reaction was determined from the initial absorbance A_0_ and the absorbance at reaction completion A_∞_. Pseudo-first-order rate constants (*k*) ware calculated from the expression t_1/2_ = ln2/k.

## 3. Results

### 3.1. Identification of the Extract Components

**Figure 2:**
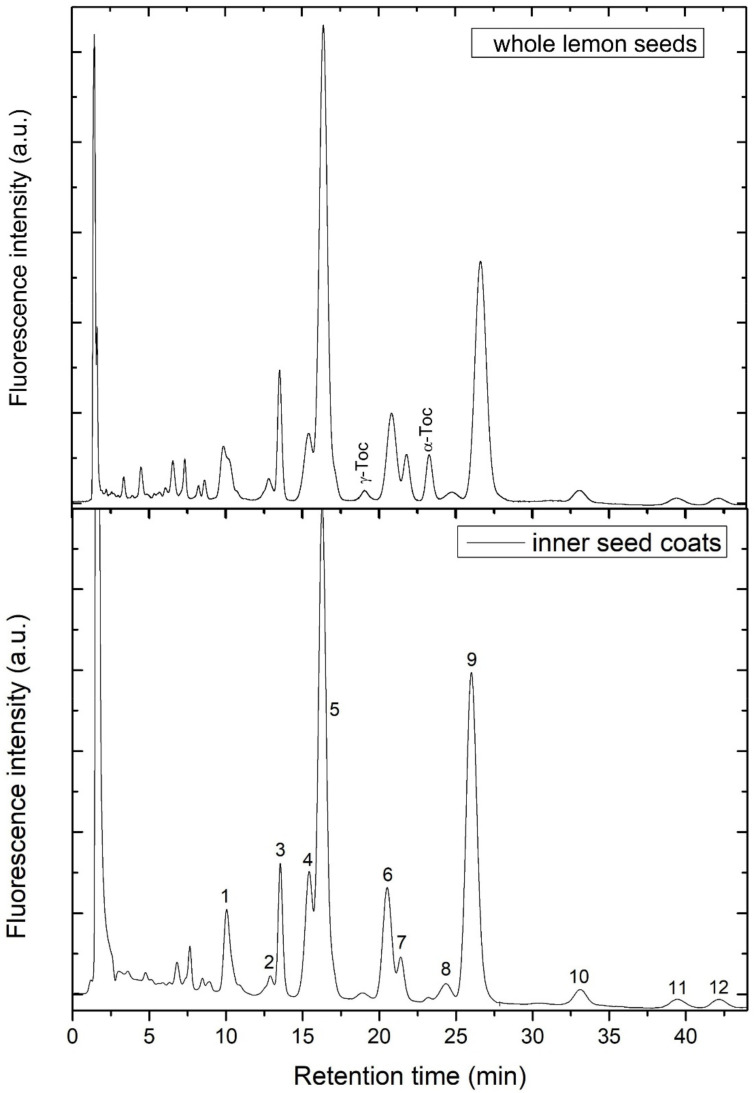
HPLC chromatograms of the extract from whole lemon seed (**top**) and inner seed coats (**bottom**). Further details are given in the Materials and Methods section.

**Figure 3:**
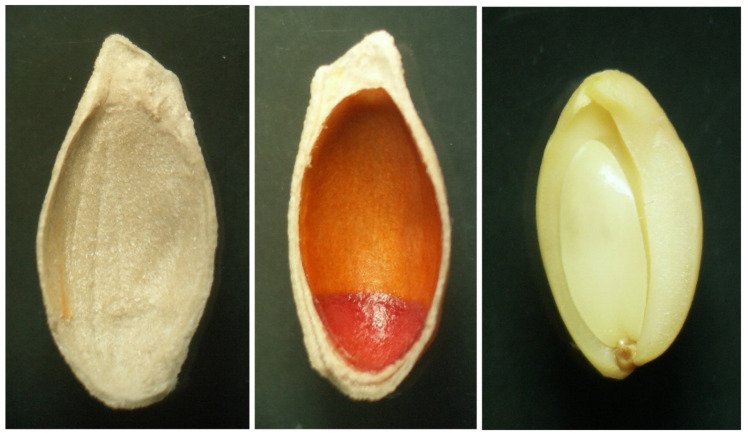
Photographs of the outer seed coat (**left**), inner seed coat (orange) (**middle**) and endosperm (**right**) of the lemon seed.

In the extract of whole lemon seeds, besides minor peaks corresponding to tocopherols, we found several other peaks in the chromatogram using fluorescence detection (Figure 2). In the extract of inner seed coat of lemon (Figure 2 and Figure 3) all these peaks (1–12), besides those of tocopherols, were found again at similar proportions as in the extract of whole seeds. When outer seed coats and endosperm (Figure 3) were analyzed separately (Figure S2), only small amounts of the unknown compounds were detected, but relatively higher content of tocopherols was found. Quantitatively, the content of the unknown compounds in the outer seed coat and the endosperm was only 3.4 and 2% of that found in the inner seed coat, respectively.

**Figure 4:**
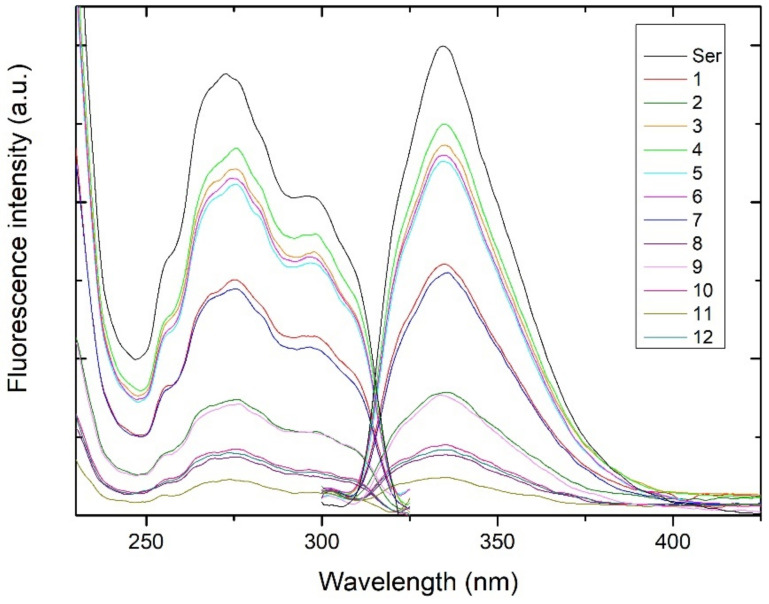
Fluorescence excitation and emission spectra of serotonin and the isolated compounds 1–12 from lemon seeds in the HPLC solvent. Further details are given in the Materials and Methods section.

To investigate these compounds in detail, we applied preparative isolation of the extract from the inner seed coats of 130 lemon seeds, column chromatography on neutral alumina and preparative HPLC chromatography. The isolated fractions corresponding to peaks 1–12 were first analyzed by fluorescence excitation and emission spectroscopy. The shape and maxima of excitation and emission spectra of the investigated compounds were very similar to each other and strongly resembled those of serotonin (Figure 4, Table 1) and recently reported acylserotonins found in baobab seed oil [11]. The best fit of the *m/z* data for the analyzed compounds obtained by HR–MS spectrometry led to number of homologues differing in hydrocarbon chain-length as Na^+^ adducts (Table 1), which are most probably *N*-acylserotonin homologues, identical or similar to those found previously [11]. Many of the analyzed compounds showed nearly the same *m*/*z* ratio, thus the same molecular formula and are supposed to be isomers (Table 1). 

**Figure 5:**
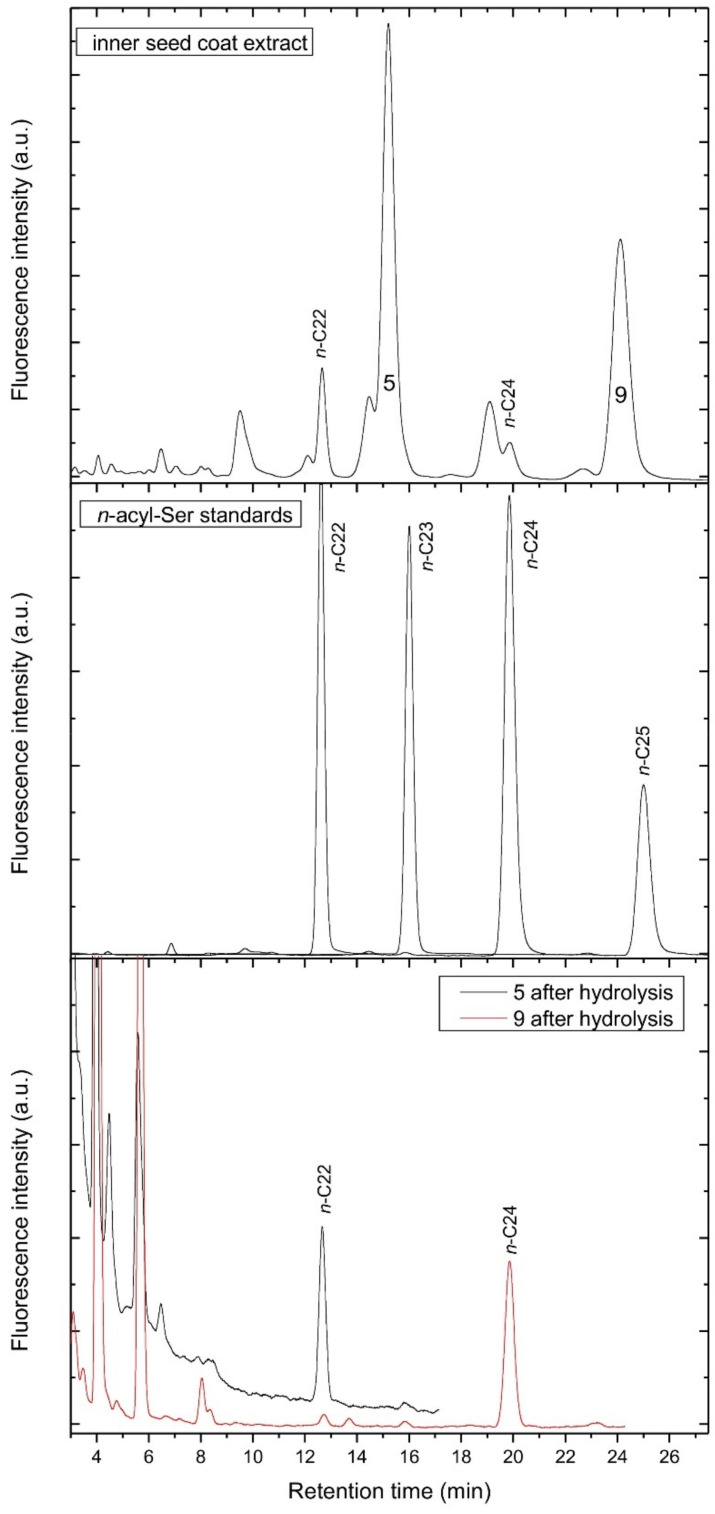
HPLC chromatograms of the inner seed coat extract of lemon (**top**), *n*-acyl-serotonin standards (**middle**) and acylserotonins obtained from fatty acids released from compounds 5 and 9 after acid hydrolysis (**bottom**).

**Figure 6:**
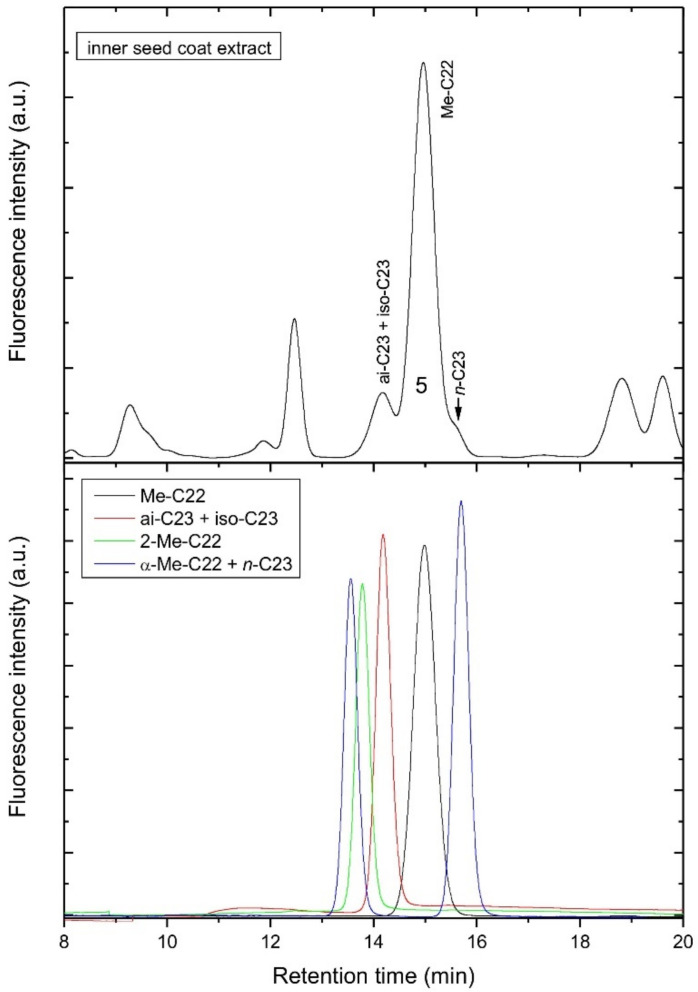
HPLC chromatograms of the inner seed coat extract of lemon (**top**) and various C23-serotonin and C22-methylserotonin standards (**bottom**). Further details are given in the Materials and Methods section.

As synthetic normal-chain acylserotonins did not match the retention time of the most abundant compounds 5 and 9, but only minor peaks corresponding to *n*-C22 and *n*-C24 serotonins in the inner seed coat extract (Figure 5), the compounds 5 and 9 must be isomers of *n*-C23 and *n*-C25 serotonins, respectively. Taking into consideration the fact that in lemon and other citrus seeds, *iso* and *anteiso* fatty acids were found [14], we originally supposed that the compounds 5 and 9 were the *iso* or *anteiso* acylserotonins. However, as shown in Figure 6, the retention time of iso-C23 and ai-C23, with the same retention time, is evidently shorter than that of compound 5 and corresponded to a minor peak in the chromatogram of the extract (Figure 6). In order to reveal the fatty acids of compounds 5 and 9, they were hydrolyzed and coupled synthetically to serotonins. The obtained compounds corresponded to n-C22 and n-C24 serotonins, respectively (Figure 5). This suggested that the compounds 5 and 9 are normal chain acyl-methylserotonins where the additional methyl group is located in the serotonin moiety. Among such serotonins, 2-methyl-, α-methyl- and *N*ω-methylserotonins were commercially available and were used for the synthesis of the corresponding acylserotonins. As shown in Figure 6, only *N*ω-methyl-C23-serotonin matched the compound 5 in the chromatogram, indicating that we deal with this compound in the extract. 

**Figure 7:**
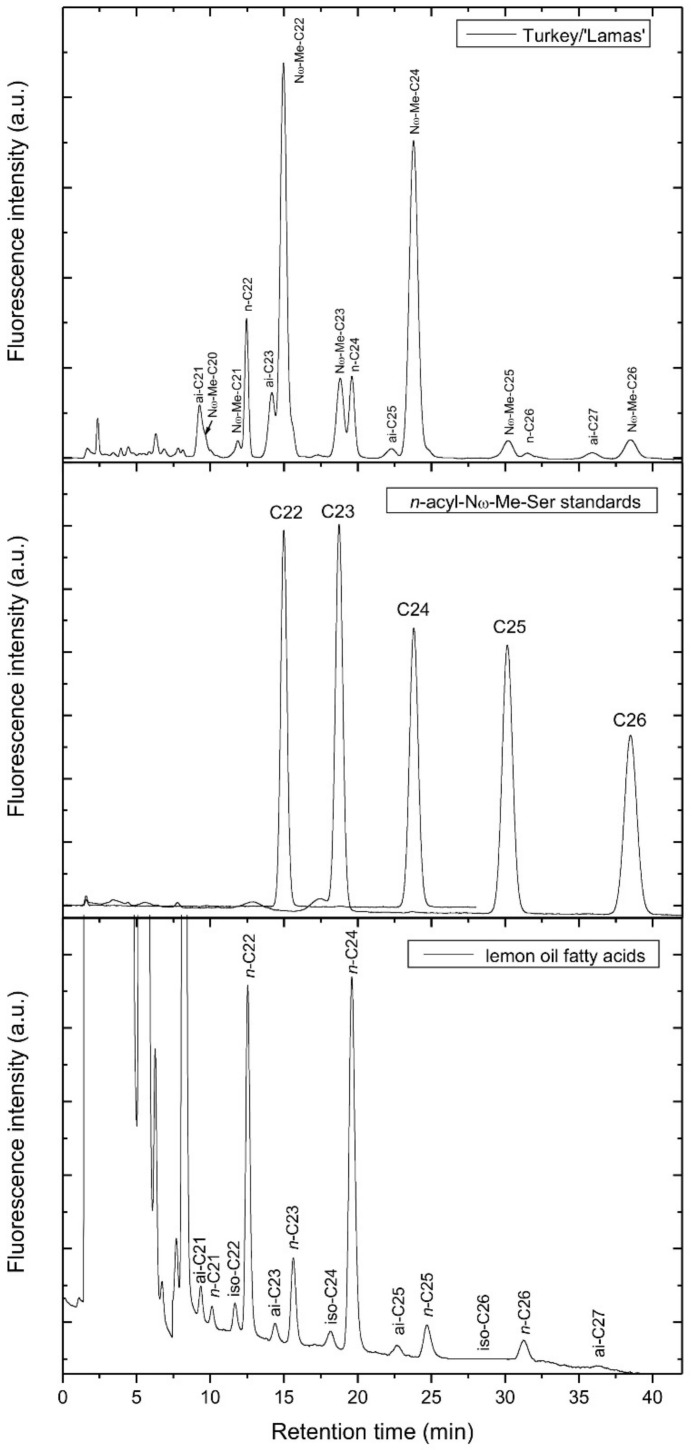
HPLC chromatograms of the extract from inner seed coats of lemon (**top**), *n*-acyl-*N*ω-methylserotonin standards (**middle**) and acylserotonins obtained from fatty acids released from lemon seed oil after hydrolysis (**bottom**). Further details are given in the Materials and Methods section.

Next, we synthesized a series of *n*-acyl-*N*ω-methylserotonins homologues and compared their retention time with those of the peaks in the extract (Figure 7). As can be seen, the standards matched well the most abundant compounds in the extract. To obtain a series of iso and *anteiso* acylserotonin homologues, a sample of lemon seed oil was hydrolyzed and the released fatty acids were coupled to serotonin. As lemon seeds are known to contain low amounts of branched fatty acids (iso with even carbon atom number, *anteiso* with an odd carbon atom number) [14], lemon seed oil was a convenient source for preparation of standards (Figure 7). A similar pattern of branched fatty acid-serotonins was obtained from lanolin (data not shown), which is a rich source of iso and *anteiso* fatty acids [15]. All of these results let us to identify all the peaks in HPLC chromatograms of the inner seed coat extract of lemon (Figure 6). Since iso and *anteiso* fatty acid isomers of acylserotonins are not separated under the applied HPLC conditions (Figure 6), their assignment in the seed extract was based on the fatty acid composition of the seed oil (Figure 7).

### 3.2. Composition of N-acylserotonins in Seeds of Different Lemon Varieties and Other Citrus Species

**Figure 8:**
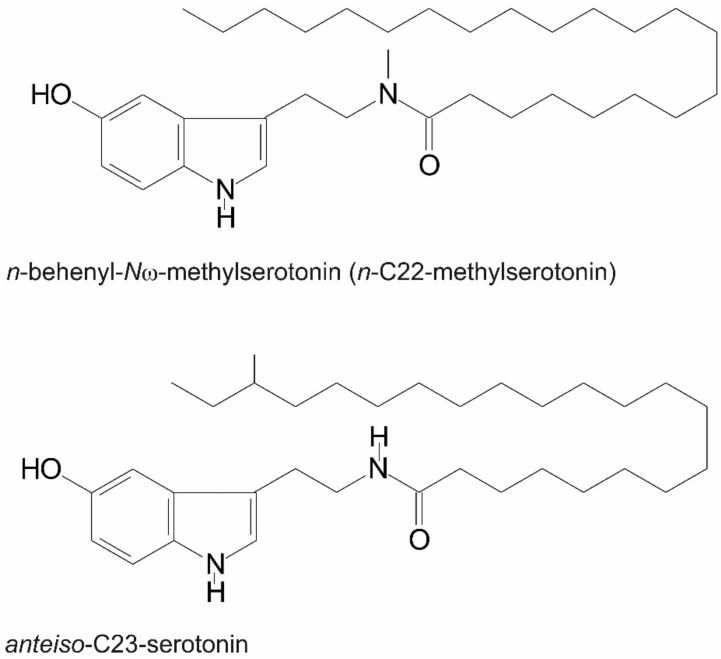
Chemical structure of *n*-behenyl-*N*ω-methylserotonin (*n*-C22-methylserotonin) and *anteiso*-C23-serotonin.

Quantitative analysis of the composition of acylserotonins in the inner seed coat of the lemon variety ’Lamas’ (Table 1) shows that long-chain acyl-methylserotonins strongly dominate (nearly 80%), among which methyl-C22-serotonin (Figure 8) and methyl-C24-serotonin are the most abundant compounds, accounting for 38.6 and 26.8%, respectively. Normal chain acylserotonins are minor constituents (8.8%). Moreover, several branched-chain, *anteiso* acylserotonins were found at low amounts with ai-C23 acylserotonin (Figure 8) as the main component (5.8%). The variety ‘Primaflora’ showed similar composition of acylserotonins (Table 2, Figure S3). The most pronounced difference was the lower content of *n*-C22-serotonin (Table 2). In contrast to these varieties, ‘Verna’ contained considerably more normal-chain acylserotonins (%) at the expense of the methylserotonin derivatives, which accounted for 45% in this case (Table 2, Figure S3). The content of total acylserotonins in the inner seed coat was similar for the investigated varieties and accounted for 1.02–1.54 mg/g fresh weight of the coats. These contents are considerably higher than those of acylserotonins in the baobab seed oil, which accounted for up to 0.3 mg/g oil [13]. It should be stressed that besides normal-chain acylserotonins, all the other compounds identified in the present studies were unknown so far.

To investigate if acylserotonins accumulate in other parts of the lemon plant, we analyzed lemon seedlings in this respect. It was found that acylserotonins (Me-C22 and Me-C24) occurred only at trace amount, together with comparable content of tocopherols, both in the primary leaves and stems of the seedlings (Figure S4). In adult lemon leaves, acylserotonins were not detected. Interestingly, acylserotonins were also not found in lemon seed oil (data not shown).

Next, we analyzed seeds of other representatives of the genus *Citrus*, such as tangerine, grapefruit and pomelo for the presence of acylserotonins. The HPLC chromatograms of whole seed extracts of these species and percent composition of the identified compounds are shown in Figure S5 and Table 3, respectively. Seeds of all of the three species contained acylserotonins identified previously in lemon and some tocopherols. In the case of tangerine and grapefruit, the composition of acylserotonins was similar to that of lemon (Table 3) with the domination of Me-C22 and Me-C24-serotonins and over 80% of total acyl-methylserotonins among the investigated compounds. On the other hand, the composition of pomelo seeds resembled that of the lemon variety ’Verna’ where normal chain acylserotonins are found at the highest level (Table 3). 

### 3.3. Antioxidant Activity of N-acylserotonins

To investigate the antioxidant efficiency of selected, newly described *N*-acylserotonins, we determined the half-times of the reaction and pseudo-first order reaction rate constants in their reaction with a stable radical DPPH in two solvents of different polarity, i.e., hexane and acetonitrile (Table 4), whose polarity corresponds to different regions of natural membranes. For comparison we included α- and δ-tocopherols (Toc). In the investigated reaction, Me-C22 and Me-C24-serotonins were less active in hexane than *n*-C22 and *n*-C24-serotonins and δ-tocopherol, while in acetonitrile all these compounds showed similar and considerably lower antioxidant activity (Table 4). On the other hand, branched-chain acylserotonins (iso-C21 and -C25) showed higher antioxidant activity than normal chain compounds (n-C22 and -C24) and Me-C22 and C24-serotonins in hexane but similar activity in acetonitrile. Generally, in the case of all of the investigated tocopherols and *N*-acylserotonins, their antioxidant activity was considerably higher in the hydrophobic solvent (hexane) than in the polar medium (acetonitrile). A similar correlation was previously observed for the reaction of substituted phenoxyl radical with biological hydroquinones [16] and phenolic prenyllipids with DPPH [13].

## 4. Discussion

In the present studies we have found a number of previously unknown acylserotonins, whose occurrence and most probably biosynthesis is confined to the inner seed coat of lemon and other citrus species. This is in contrast to recently identified acylserotonins which were found in seed oil [11] which suggests that their synthesis proceeds in endosperm.

Normal chain acylserotonins, identified recently in baobab seed oil [11], were found in citrus seeds as minor components in most cases. Unexpectedly, the dominant compounds were acyl-derivatives of *N*ω-methylserotonin, which is very rarely found in plants, in contrast to serotonin [17,18]. *N*ω-methylserotonin, with the methyl group located at the amino group of the side-chain, was found so far in *Hordeum vulgare*, *Desmodium* species, *Piptadenia* sp. [19], *Cimicifuga racemosa* [20] and recently in lemon and other citrus species [21] besides *N,N*-dimethyl-, *N,N,N*-trimethylserotonin and the corresponding glucosides [22]. The glucosides were also identified in *Zanthoxylum piperitum* seeds [23]. In bergamot (*Citrus bergamium*) seeds, the content of *N*-methylserotonin was considerably higher than that of serotonin [21]. If this is the case for the ‘Lamas’ and ‘Primaflora’ lemon varieties and tangerine, as well as grapefruit seeds, it explains the higher content of acyl-methylserotonins than that of acylserotonins in these seeds, assuming that the enzyme engaged in acylserotonin synthesis uses both serotonin and methylserotonin as substrates with similar yields. In the variety ‘Verna’ and pomelo seeds, the proportion of methylserotonin and serotonin is probably similar, resulting in approximately equal acylserotonins and acyl-methylserotonins (Table 2 and Table 3). 

Among previously unknown acylserotonins, we identified branched chain serotonin derivatives. Such fatty acids have been known for a long time to exist in bacteria [24] and are also found in milk [25] and lanolin [15]. In plants, these fatty acids are rarely found and always in minor amounts [26,27]. In the Plant Fatty Acids Database [27], there are 133 species (47 of angiosperms, 86 of conifers) reported to contain branched fatty acids—most of them have *anteiso*-C17 (0.1–1.9% of fatty acids), followed by iso-C17 (0.4–1.7%) and single species with iso-C14, -C15, -16, -18, *anteiso*-C15 and -C16. Interestingly, lemon and other citrus species are the only species reported so far to have long-chain branched fatty acids in fruits and seeds (up to C26) [14]. The content of branched fatty acids reached 7.8% and 0.57% of total fatty acids in juice and seeds, respectively. This explains the presence of branched-chain acylserotonins in citrus seeds. The composition of acylserotonins in terms of fatty acid chain length corresponds to the long-chain fatty acid composition of seeds (Figure 7 and [14]). This indicates that the enzyme synthesizing the acylserotonins shows no preference for odd-carbon-number fatty acids, as it was the case of baobab. On the other hand, similarly as in baobab, the enzyme must have a strong preference for long-chain, saturated fatty acids as these acids are very minor components of citrus seeds [14] (*n*-C22 and *n*-C24 constitute 0.12 and 0.067% of fatty acids in lemon seeds, respectively) and no acylserotonins were found with the most abundant fatty acids of lemon seeds, such as linoleic, oleic, palmitic or linolenic acids [16]. It is known that besides long-chain hydrocarbons, aldehydes, primary alcohols and fatty acids are also constituents of fruit and leaf cuticular waxes which is also the case for several citrus species [28]. While in hydrocarbon fractions of lemon, orange, clementine and mandarin waxes, odd-carbon- number hydrocarbons prevailed (C29, C31 and C33), even-carbon-number compounds were the most abundant in the fractions of the other compounds (C24 to C32) [28]. Among fatty acids of cuticular wax of lemon fruits, the main fatty acid was melissic acid (C30), followed by C32, C28 and C24 acids. This indicates that determination of the chain length of fatty acids used for the biosynthesis of *N*-acylserotonins in the inner seed coat of citrus species relies on different mechanisms than in the case of fatty acids of seed triacylglycerols and those of cuticular waxes. 

As in the case of acylserotonins found in baobab seed oil [11], the selected compounds identified for the first time in the present studies were shown to have antioxidant activity in vitro, similar to that of δ-tocopherol. The antioxidant activity of the investigated compounds might be the primary function in the inner seed coats of citrus fruits. 

Moreover, taking into account the fact that various synthetic serotonin analogues are used as serotonin agonists [29,30], the natural acyl-methylserotonins could find similar pharmacological applications. 

## Figures and Tables

**Table 1:** Fluorescence excitation and emission maxima of the isolated compounds 1–12 (Figure 2) from inner seed coats of lemon and HR–MS data of these compounds. Further details are given in the Materials and Methods section. sh—shoulder.

Peak Number	Fluorescence Maxima (nm)		HR–MS (*m*/*z*)	Molecular Formula	Exact Mass	Δ (mDa)	Corresponding Structure
	Excitation	Emission					
1	275, 298 sh	335	507.3921	C_31_H_52_N_2_O_2_Na^+^	507.3921	0.0	C21-Ser
2	275, 298 sh	335	521.4081	C_32_H_54_N_2_O_2_Na^+^	521.4078	0.3	C22-Ser
3	275, 298 sh	335	521.4073	C_32_H_54_N_2_O_2_Na^+^	521.4078	0.5	C22-Ser
4	275, 298 sh	335.5	535.4235	C_33_H_56_N_2_O_2_Na^+^	535.4234	0.1	C23-Ser
5	275, 298 sh	335	535.4227	C_33_H_56_N_2_O_2_Na^+^	535.4234	0.7	C23-Ser
6	274.5, 298 sh	335	549.4384	C_34_H_58_N_2_O_2_Na^+^	549.4391	0.7	C24-Ser
7	275, 298 sh	335.5	549.4386	C_34_H_58_N_2_O_2_Na^+^	549.4391	0.5	C24-Ser
8	275, 298 sh	335	563.4563	C_35_H_60_N_2_O_2_Na^+^	563.4547	1.6	C25-Ser
9	275, 298 sh	334	563.4557	C_35_H_60_N_2_O_2_Na^+^	563.4547	1.0	C25-Ser
10	275, 298 sh	335	577.4699	C_36_H_62_N_2_O_2_Na^+^	577.4704	0.5	C26-Ser
11	274.5, 298.5 sh	335	591.4872	C_37_H_64_N_2_O_2_Na^+^	591.4860	1.2	C27-Ser
12	274, 298 sh	335	591.4857	C_37_H_64_N_2_O_2_Na^+^	591.4860	0.3	C27-Ser

**Table 2:** Percent composition (mol%) of *N*-acylserotonins in the inner seed coats of various lemon varieties and the content of total acylserotonins in the seed coats (means ± SE, *n*=3). Acylserotonins with the highest content are marked in bold. n.d.—not detected.

Acylserotonin	Turkey/‘Lamas’	Italy/‘Primaflora’	Spain/‘Verna’
<C21	2.3	1.7	2.9
ai-C21	3.3	0.8	0.7
Me-C20	2.2	1.0	0.4
n-C21	n.d.	n.d.	0.3
Me-C21	1.1	0.75	1.9
**n-C22**	6.0	2.25	**20.7**
ai-C23	5.8	3.1	3.7
**Me-C22**	**38.6**	**42.8**	**14.3**
n-C23	n.d.	n.d.	4.3
Me-ai-C23	0.2	0.4	n.d.
Me-C23	7.1	8.8	3.7
**n-C24**	2.8	1.8	**16.6**
ai-C25	0.8	0.6	0.5
**Me-C24**	**26.8**	**31.0**	**21.2**
n-C25	n.d.	n.d.	1.1
iso-C26	n.d.	0.14	n.d.
Me-C25	1.1	2.0	1.9
n-C26	n.d.	0.08	1.2
ai-C27	1.0	0.6	1.0
Me-C26	0.9	1.9	2.5
n-C27	n.d.	n.d.	0.1
iso-C28	n.d.	0.2	0.2
Me-C27	n.d.	0.04	0.2
n-C28	n.d.	n.d.	0.4
**total acyl-Me-Sers (%)**	**77.8**	**88.3**	**45.1**
**total n-acyl-Sers (%)**	**8.8**	**4.1**	**44.7**
**total acyl-Sers** **mg/g FW**	**1.15 ± 0.16**	**1.54 ± 0.32**	**1.02 ± 0.24**

**Table 3:** Percent composition (mol%) of *N*-acylserotonins in seeds of various citrus species. Acylserotonins with the highest content are marked in bold. n.d.—not detected.

Acylserotonin	Tangerine	Grapefruit	Pomelo
<C21	2.7	2.8	2.1
ai-C21	1.3	1.3	1.5
Me-C20	1.1	1.0	n.d.
n-C21	0.1	n.d.	0.1
Me-C21	0.6	0.7	0.3
**n-C22**	3.3	4.1	**18.5**
ai-C23	3.6	3.1	n.d.
**Me-C22**	**40.5**	**36.1**	**14.6**
n-C23	n.d.	1.1	6.8
Me-C23	9.7	9.8	6.0
**n-C24**	4.3	3.7	**21.8**
ai-C25	n.d.	0.9	n.d.
**Me-C24**	**29.0**	**30.5**	**22.5**
n-C25	n.d.	1.1	2.1
iso-C26	n.d.	n.d.	n.d.
Me-C25	3.1	2.6	1.3
n-C26	0.5	0.3	1.0
ai-C27	n.d.	0.8	1.3
**total acyl-Me-Sers (%)**	**83.0**	**81.6**	**44.7**
**total n-acyl-Sers (%)**	**8.2**	**10.3**	**50.3**

**Table 4:** Half-times of the reaction and pseudo-first-order reaction rate constants k for the reaction of *N*-acylserotonins and tocopherols with DPPH in solvents of different polarity. The data are means ± SE (*n* = 3).

Compound	Hexane		Acetonitrile	
	t_1/2_ (s)	k (10^−3^ s^−1^)	t_1/2_ (s)	k (10^−3^ s^−1^)
α-Toc *	11.2 ± 0.7	61.6 ± 4.1	22.0 ± 1.0	31.6 ± 1.4
δ-Toc	26.5± 0.5	26.1 ± 0.6	268.0 ± 12.0	2.6 ± 0.1
n-C22 *	27.5 ± 3.0	25.2 ± 2.7	328 ± 114	2.1 ± 0.7
n-C24 *	28.0 ± 2.5	24.7 ± 2.2	183 ± 12	3.7 ± 0.2
Me-C22	38.0 ± 2.0	18.2 ± 0.9	404 ± 12	1.7 ± 0.1
Me-C24	43.5 ± 1.5	15.9 ± 0.6	268 ± 16	2.6 ± 0.2
iso-C21	25.1 ± 0.4	27.6 ± 0.4	366 ± 10	1.9 ± 0.1
iso-C25	17.0 ± 0.6	40.8 ± 1.4	414 ± 30	1.6 ± 0.1

* Data taken from [11].

## Data Availability

Not applicable.

## Supplementary Materials

The following supporting information can be downloaded at: https://www.mdpi.com/article/10.3390/biom12101528/s1, Figure S1: HPLC chromatograms of the extract from outer seed coat (top) and seed endosperm (bottom) of lemon seeds. In the case of endosperm, it was first extracted with the HPLC solvent (solvent A) and then with hexane. The volume of the extracts in Figure 1 (bottom) and S1 were the same, as well as the scale of the fluorescence signal, so the content of the detected compounds can be directly compared. Further details are given in the Materials and Methods section.; Figure S2: HPLC chromatograms of the inner seed coat extract of lemon varieties ‘Primaflora’ from Italy and ‘Verna’ from Spain. Further details are given in the Materials and Methods section; Figure S3: HPLC chromatograms of the extract from the primary leaves and stems of lemon seedlings. Further details are given in the Materials and Methods section; Figure S4: HPLC chromatograms of the extracts from whole seeds of tangerine, grapefruit and pomelo. Further details are given in the Materials and Methods section.

## Abbreviations

DPPH	2,2-diphenyl-1-picrylhydrazyl
EDC	*N*-(3-dimethylaminopropyl)-N′-ethylcarbodiimide hydrochloride
THF	tetrahydrofuran
DMF	dimethylformamid
HOBT	1-hydroxybenzotriazole hydrate
FW	fresh weight
Ser	serotonin
