# Supplementary material for: Acyl-Nω-methylserotonins and Branched-chain Acylserotonins in Lemon and Other Citrus Seeds—New Lipids with Antioxidant Properties and Potential Pharmacological Applications
Source: Biomolecules. 2022 Oct 20;12(10):1528. doi: 10.3390/biom12101528 (PMC9599447; doi:10.3390/biom12101528)
Supplement: Supplementary file 1 [file biomolecules-12-01528-s001.zip › biomolecules-1945043-supplementary.pdf]

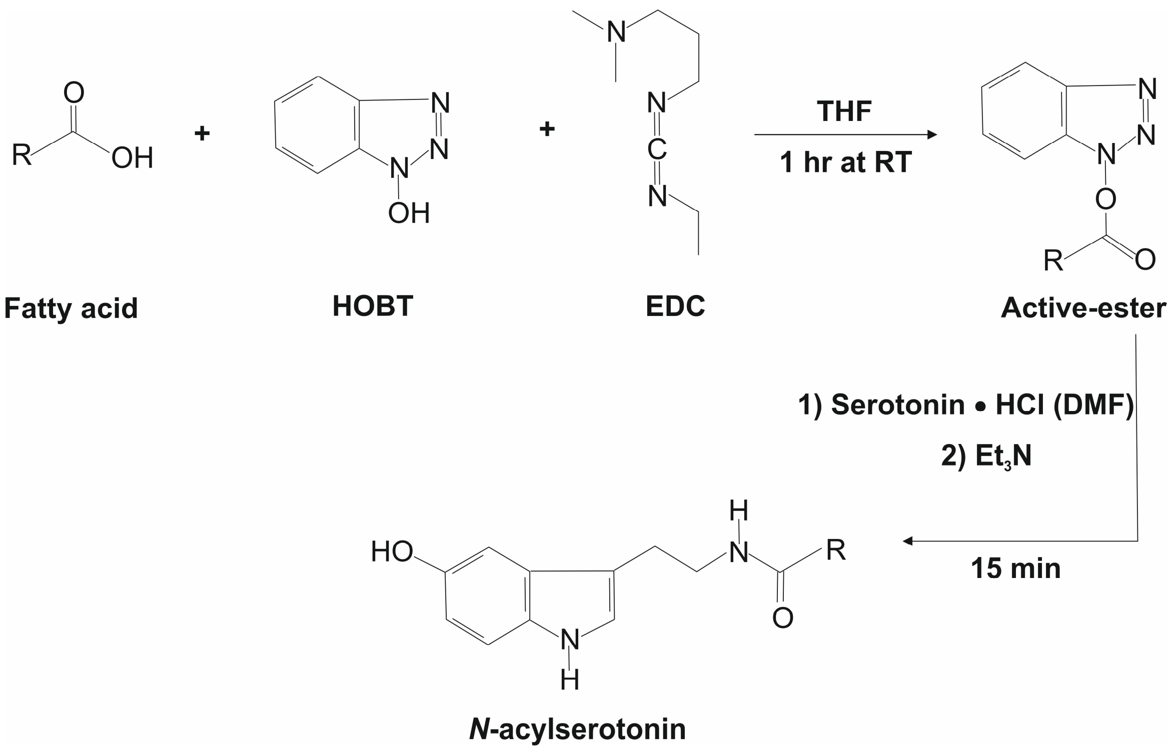

Figure S1. Synthesis of *N*-acylserotonins. R - fatty acid side-chain.

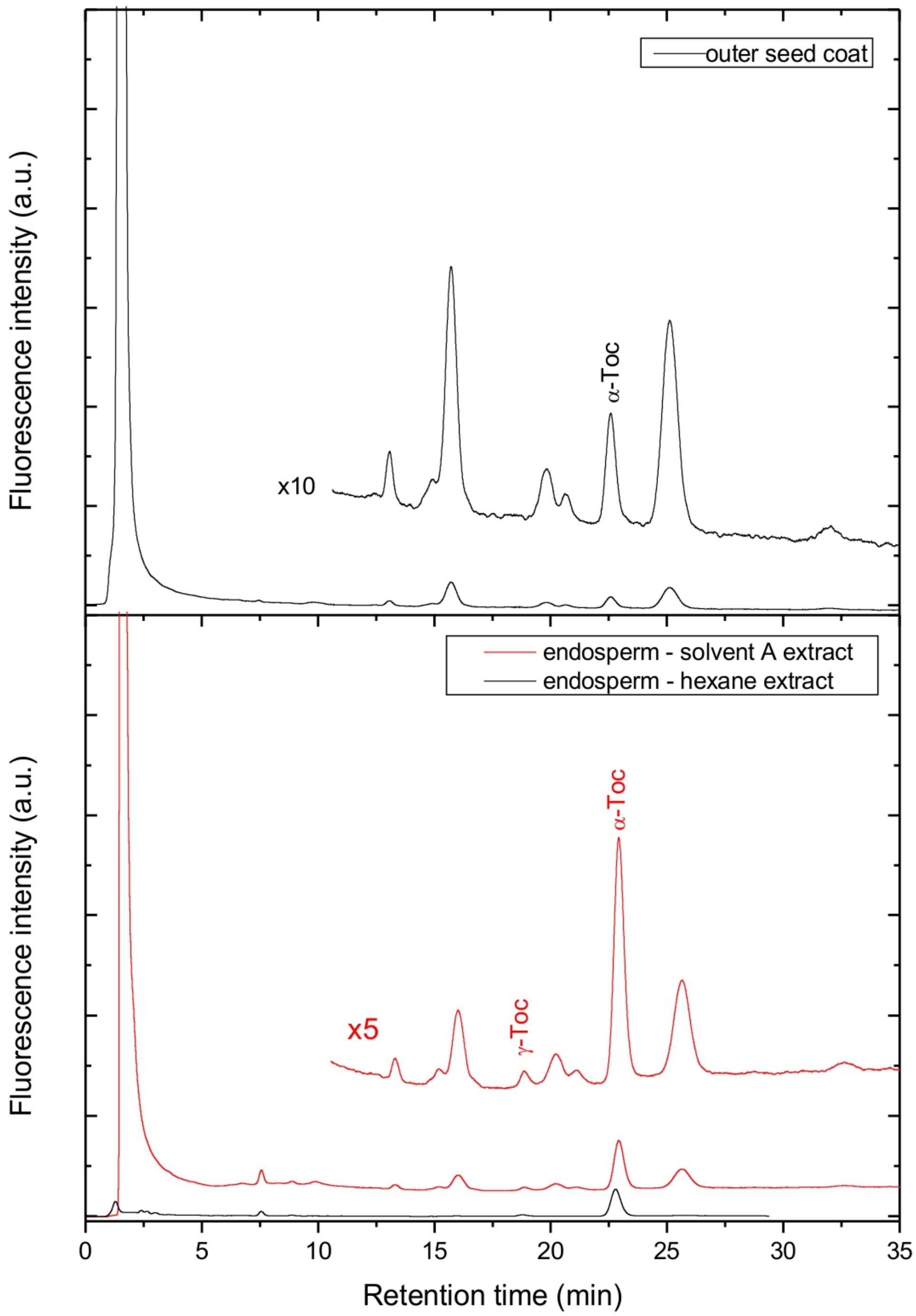

Figure S2. HPLC chromatograms of the extract from outer seed coat (top) and seed endosperm (bottom) of lemon seeds. In the case of endosperm, it was first extracted with the HPLC solvent (solvent A) and then with hexane. The volume of the extracts in Figure 2 (bottom) and S2 was the same, as well as the scale of the fluorescence signal, so the content of the detected compounds can be directly compared. Further details are given in the Experimental section.

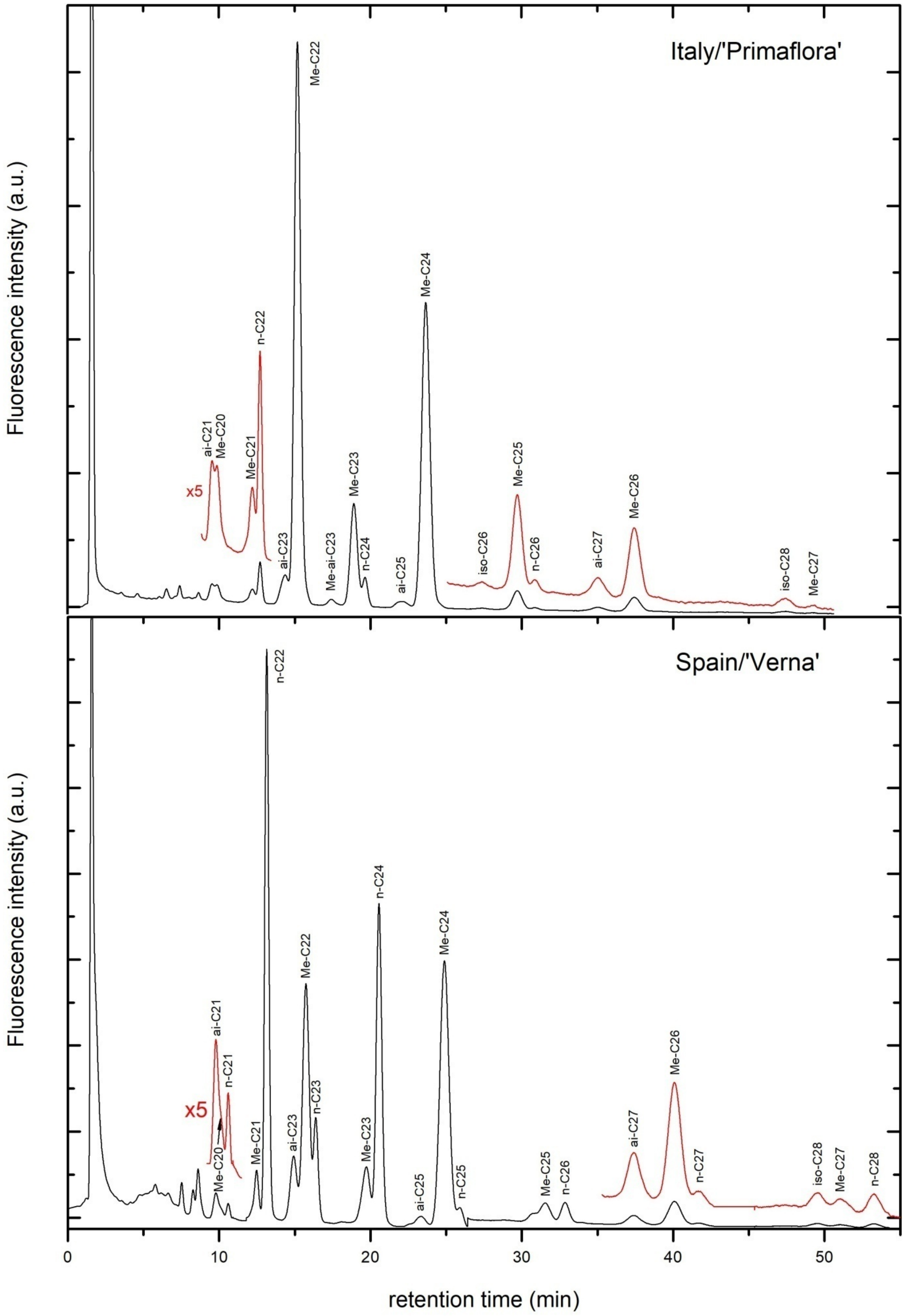

Figure S3. HPLC chromatograms of the inner seed coat extract of lemon varieties ‘Primaflora’ from Italy and ‘Verna’ from Spain. Further details are given in the Experimental section.

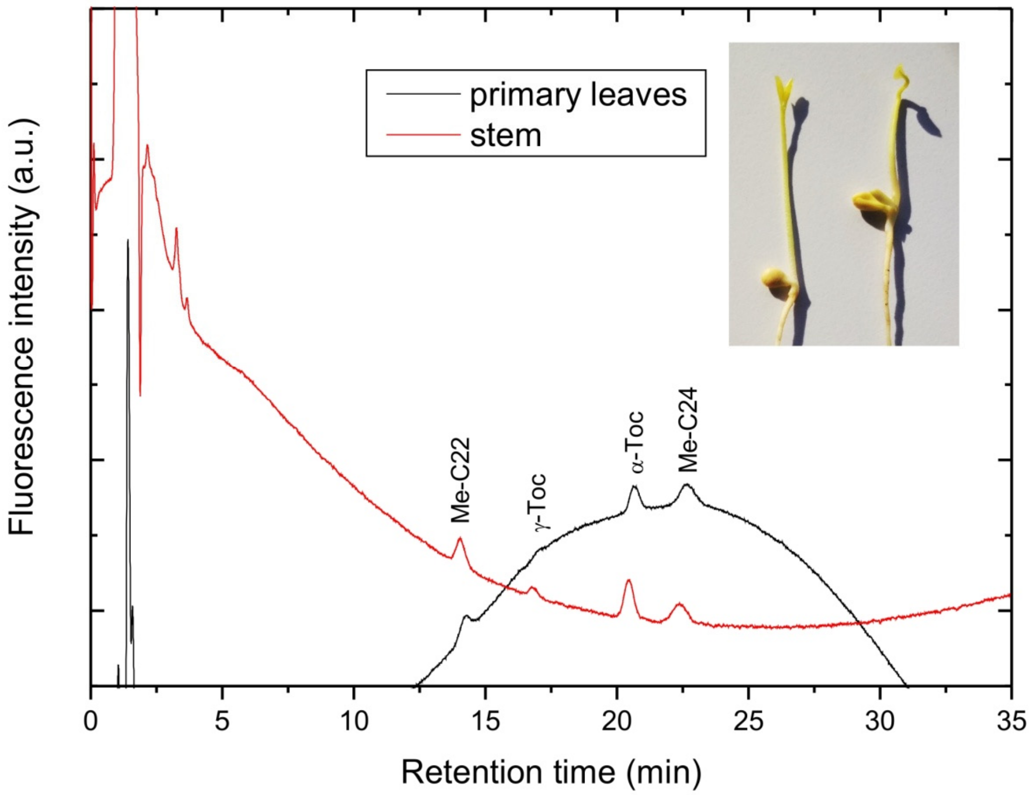

Figure S4. HPLC chromatograms of the extract from the primary leaves and stems of lemon seedlings. Further details are given in the Experimental section.

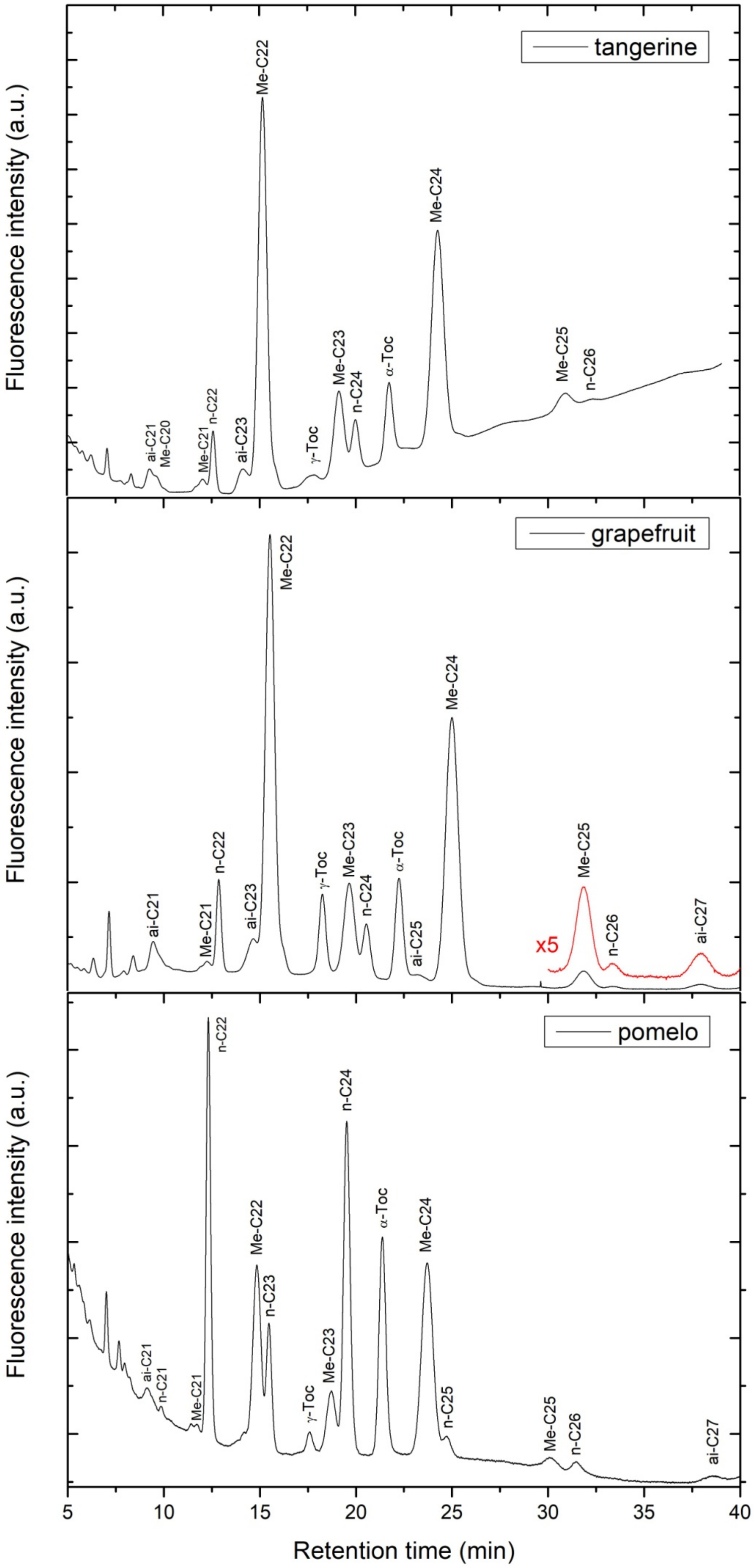

Figure S5. HPLC chromatograms of the extracts from whole seeds of tangerine, grapefruit and pomelo. Further details are given in the Experimental section.
